# Repurposing auranofin as an intestinal decolonizing agent for vancomycin-resistant enterococci

**DOI:** 10.1038/s41598-018-26674-0

**Published:** 2018-05-29

**Authors:** Ahmed AbdelKhalek, Nader S. Abutaleb, Khalifa A. Elmagarmid, Mohamed N. Seleem

**Affiliations:** 1 Department of Comparative Pathobiology, College of Veterinary Medicine, Purdue University, West Lafayette, IN 47907 USA; 2 Purdue Institute for Inflammation, Immunology, and Infectious Diseases, West Lafayette, IN 47907 USA

## Abstract

Multidrug-resistant enterococcal pathogens, especially vancomycin-resistant enterococci (VRE), are among the pathogens that require new antibiotic innovation. The colonization of the gut represents a major pathway by which VRE can cause infection and spread to other patients. In the current study, auranofin (FDA-approved rheumatoid arthritis drug) is evaluated for its potential use as a decolonizing agent for VRE. Auranofin was found to exert potent antimicrobial activity against a wide range of enterococcal clinical isolates with a minimum inhibitory concentration of 1 μg/mL. No resistant mutants could be developed against auranofin over the course of 14 passages. Auranofin was also found to exert potent anti-biofilm activity against VRE. Auranofin was superior to linezolid, the drug of choice for VRE infection treatment, in the *in vivo* mouse model. Auranofin significantly reduced the VRE burden in feces, cecum, and ileum contents after 8 days of treatment. Accordingly, this study provides valuable evidence that auranofin has significant promise as a novel gastrointestinal decolonizing agent for VRE.

## Introduction

Enterococcal species are one of the major pathogens of healthcare settings^1^. Two strains, *Enterococcus faecium* and *Enterococcus faecalis*, are of major concern. Both strains can lead to bloodstream infection, endocarditis, meningitis, urinary tract infection, and other infections^2^. *Enterococci*, especially *E*. *faecium*, exhibits intrinsic resistance against several classes of antibiotics and can also develop resistance via mutation or exogenous gene transfer, which resulted in the emergence of multi-drug resistant enterococcal strains. The most pronounced resistance is against vancomycin. Vancomycin-resistant enterococcal (VRE) infections comprise most *Enterococcus faecium* infections as well as a significant proportion of *Enterococcus faecalis* infections^1^.

The widespread use of broad-spectrum antibacterial agents has contributed to the elevated prevalence of these opportunistic pathogens and the reduction of their antibiotic susceptibility^2^. *Enterococci* are normal inhabitants of several tissues of the human body, particularly the gastrointestinal tract (GIT). In the GIT *Enterococci* remain under the control of other intestinal commensals and gut cell receptors. The administration of high antibiotic concentrations, like in hospital settings, leads to reduced populations of susceptible gut commensals, which allows *Enterococci* to overgrow^3,4^. The VRE domination of the gut’s microbial consortium can persist even after the cessation of antibiotic treatment, and is usually followed by the translocation of the antibiotic-resistant bacteria across the mucosal barrier, which causes systemic infections^4^.

An effective decolonizing agent is required to prevent systemic VRE infections and limit the VRE endemicity in healthcare settings^5^. Unfortunately, no drugs are approved by the Food and Drug Administration (FDA) to decolonize VRE from the intestine. The clinical molecule, ramoplanin, was evaluated in Phase II of clinical trials and was able to temporarily suppress VRE colonization and was not tested further in Phase III^6,7^.

Repurposing FDA-approved drugs for which human safety, bioavailability, and efficacy have already been proven is an efficient approach to drug discovery^8–10^. Auranofin is an FDA-approved drug for the treatment of rheumatoid arthritis, it has a well-studied safety profile^11^. Adverse effects associated with auranofin administration are rare and mostly associated with long-term use. These adverse reactions include diarrhea (2–5% of the patients), skin rash, and extremely rare thrombocytopenia. In most cases, these adverse effects were all mild and usually self-limiting^12^. We recently showed that auranofin exerts broad-spectrum antibacterial and antifungal activities^13,14^. Auranofin was also shown to possess antiprotozoal activity, and is currently in Phase II studies for the treatment of amoebic dysentery and giardiasis^15,16^. Even though auranofin is oral bioavailable FDA-approved drug, only 20–30% of the oral dose is being absorbed^17^. These characteristics make auranofin a potential candidate to decolonize VRE in the GI tract. In the current study, auranofin is being evaluated for its ability to decolonize VRE in the GI tract.

## Results

### Susceptibility of enterococcal isolates to auranofin

We tested the activity of auranofin against a wide panel of enterococcal isolates and compared auranofin to the control antibiotics. Using the standard microdilution assay, auranofin was found to exhibit potent activity against the 27 tested isolates with an MIC range of 0.5 to 1 µg/ml (Table 1). Linezolid showed an MIC range of 0.5 to 16 µg/ml, ramoplanin showed an MIC range of 0.25 to 4 µg/ml, and vancomycin showed an MIC range of 0.5 to >128. The MIC90 (the minimum inhibitory concentration that inhabited 90% of the strains) of auranofin was found to be 1 µg/ml. The MIC90 for linezolid and ramoplanin was 2 and 4 µg/ml, respectively.

**Table 1:** The minimum inhibitory concentration (MIC, µg/mL) of auranofin and control antibiotics against VRE isolates used in the study.

Strains	MIC µg/mL				Source and comments
	Auranofin	Linezolid	Ramoplanin	Vancomycin	
*E*. *faecalis* NR 31971	1	1	2	64	Urine sample obtained in Michigan, USA. Resistant to vancomycin.
*E*. *faecium* NR 31914	1	1	2	>128	Isolated in 1995 from ascites fluid of a hospitalized patient in the Netherlands.
*E*. *faecium* HM 968	1	1	1	>128	Isolated from human oral sputum collected in Colombia, 2006.
*E*. *faecalis* NR 31972	1	1	4	>128	Isolated in 2003 from a human urine sample obtained in Michigan, USA.
*E*. *faecium* NR 28978	1	1	2	>128	Hospitalized person free of enterococcal infection in the Netherlands in 2000 during a hospital surveillance.
*E*. *faecium* NR 31903	1	16	2	>128	Isolated from the stool of a human patient prior to bacteremia.
*E*. *faecium* NR 31909	1	1	4	>128	Isolated from the stool of a human patient prior to bacteremia.
*E*. *faecium* NR 31912	1	1	2	>128	Isolated from the stool of a human patient having dominance of VRE in the stool but no bacteremia.
*E*. *faecium* NR 31915	0.5	1	1	>128	Isolated in 1996 from turkey feces in the Netherlands. Resistant to gentamicin.
*E*. *faecium* NR31916	0.5	1	1	128	Isolated in 1996 from turkey feces in the Netherlands.
*E*. *faecium* NR 32052	1	1	0.5	>128	Isolated in 2008 from swine feces in Michigan, USA. Resistant to erythromycin and tetracycline.
*E*. *faecium* NR 32053	1	1	0.25	>128	Isolated in 2008 from swine feces in Michigan, USA. Resistant to erythromycin and tetracycline.
*E*. *faecium* NR 32054	0.5	1	0.25	128	Isolated in 2008 from swine feces in Michigan, USA. Resistant to erythromycin and tetracycline.
*E*. *faecium* NR 32065	0.5	1	2	>128	Isolated in 1994 in Aix-en-Provence, France.
*E*. *faecium* NR 32094	1	0.5	2	>128	Isolated in 1996 in New York, USA.
*E*. *faecium* HM 952	1	1	2	>128	Human isolate from the United States.
*E*. *faecium* HM 965	1	1	1	>128	Human blood in Ecuador, 2006. Resistant to ampicillin, gentamycin and streptomycin.
*E*. *faecium* HM 970	1	1	0.5	>128	Human feces collected in Colombia, in 2008. Resistant to vancomycin.
*E*. *faecium* ATCC 700221	1	1	4	>128	Human feces, Connecticut. Resistant to Vancomycin and Teicoplanin.
*E*. *faecalis* HM 201	1	1	2	>128	Isolated in 2002 from the blood of a patient with endocarditis at Stamford Hospital in Connecticut, USA.
*E*. *faecalis* HM 334	1	1	2	>128	Isolated in 2004 from the blood of a 64-year-old female hemodialysis patient with fatal bacteremia.
*E*. *faecalis* HM 335	1	1	2	>128	Isolated in 2004 from the blood of a 64-year-old female hemodialysis patient with fatal bacteremia.
*E*. *faecalis* HM 934	1	1	4	>128	Isolate from a human secretion in Bogota, Colombia, in 2006.
*E*. *faecium* NR 31933	1	2	2	4	Isolated in 2001 from the feces of a miniature pig in Germany.
*E*. *faecium* NR 31935	1	2	2	≤1	Isolated in 1956 from cheese in Norway.
*E*. *faecium* NR 31937	1	1	2	2	Isolated in 1957 from the blood of a hospitalized patient in the Netherlands.
*E*. *faecium* NR 31954	1	1	2	2	Isolated in 2006 from the blood of a hospitalized patient in the Netherlands.
MIC_90_	1	2	4	>128	

### Killing kinetics of auranofin

**Figure 1:**
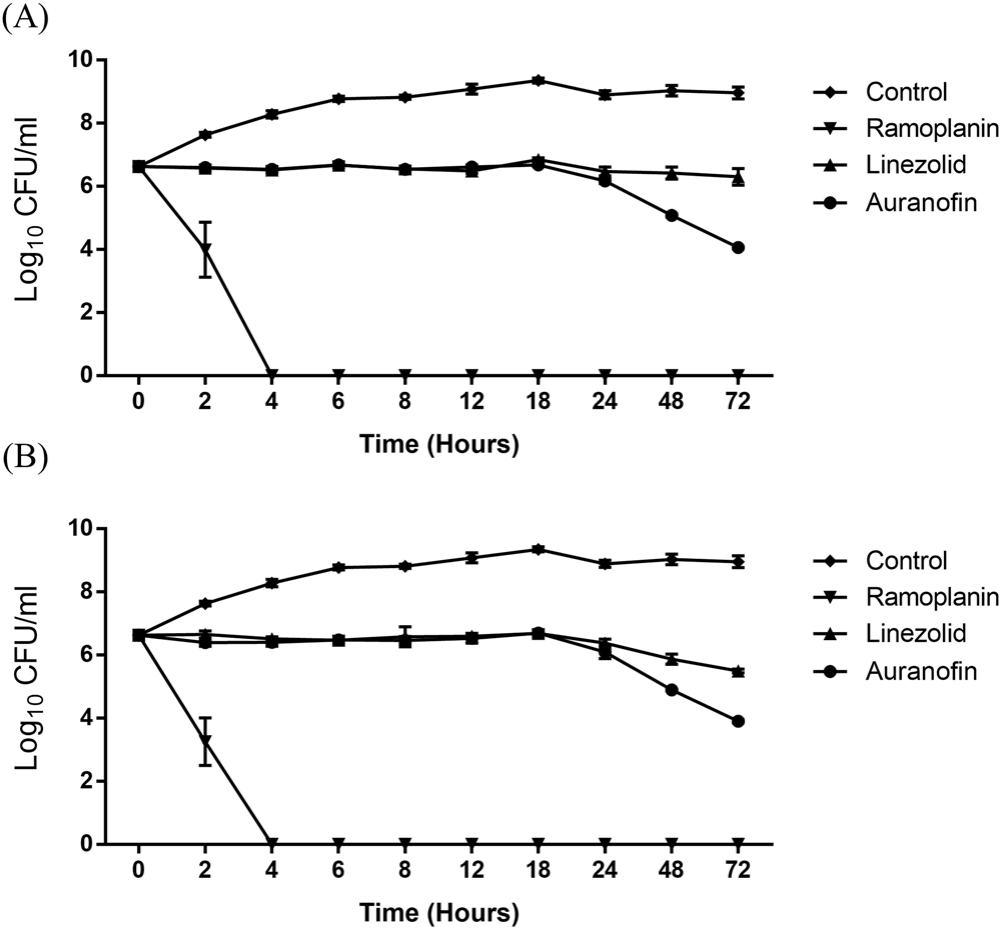
Time-kill assay for Auranofin linezolid, ramoplanin, tested at (**A**) 3 × MIC and (**B**) 6 × MIC. *E*. *faecium* HM 952 was aerobically incubated with the indicated concentrations of the drugs, in triplicates, for 72 hours at 37 °C and samples were counted at the indicated time points.

After confirming the potent activity of auranofin against VRE, we next assessed the growth kinetics of VRE strain *E*. *faecium* HM 952 when exposed to auranofin at two different concentrations: 3X and 6X the MIC. As depicted in Fig. 1, auranofin at 3X and 6X reduced VRE by 2.57 and 2.72 after 72 hours, respectively. Linezolid, a known bacteriostatic against VRE, as expected did not reduce CFU after 72 hours of exposure. Ramoplanin, a known rapid bactericidal^18,19^, demonstrated rapid bactericidal activity against VRE and cleared the VRE within 4 hours.

### *In vitro* multistep resistance development of VRE against auranofin

**Figure 2:**
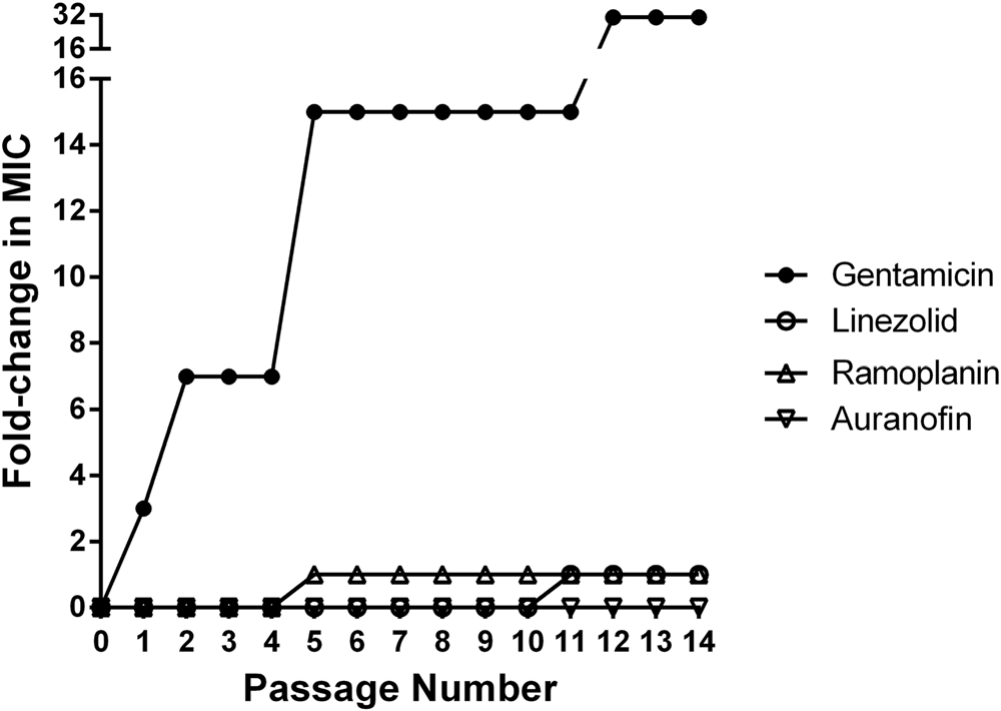
Multi-step resistance selection of auranofin, gentamicin, linezolid, and ramoplanin against VRE strain *E*. *faecium* HM 952. VRE was serially passaged with drugs for 14 days and the broth microdilution assay was used to determine the minimum inhibitory concentration of each drug against VRE after each successive passage. A 4-fold increase in the MIC is considered resistance.

In order to test the likelihood of *enterococci* to develop resistance against auranofin, we serially passaged VRE isolate *E*. *faecium* HM 952 daily in the presence of a subinhibitory concentration of auranofin and control antibiotics for 14 days^20^. A four-fold shift in the MIC was considered resistance^21^. As presented in Fig. 2, the MIC of auranofin did not change over 14 passages, which indicates that VRE did not develop resistance to auranofin. There was a one-fold increase in the linezolid’s MIC observed over 14 passages. There was a rapid shift and increase to a 7-fold MIC after the second passage for gentamicin. VRE developed a 32-fold increase in MIC to gentamicin after 14 passages with the drug.

### Activity of auranofin against VRE biofilm

**Figure 3:**
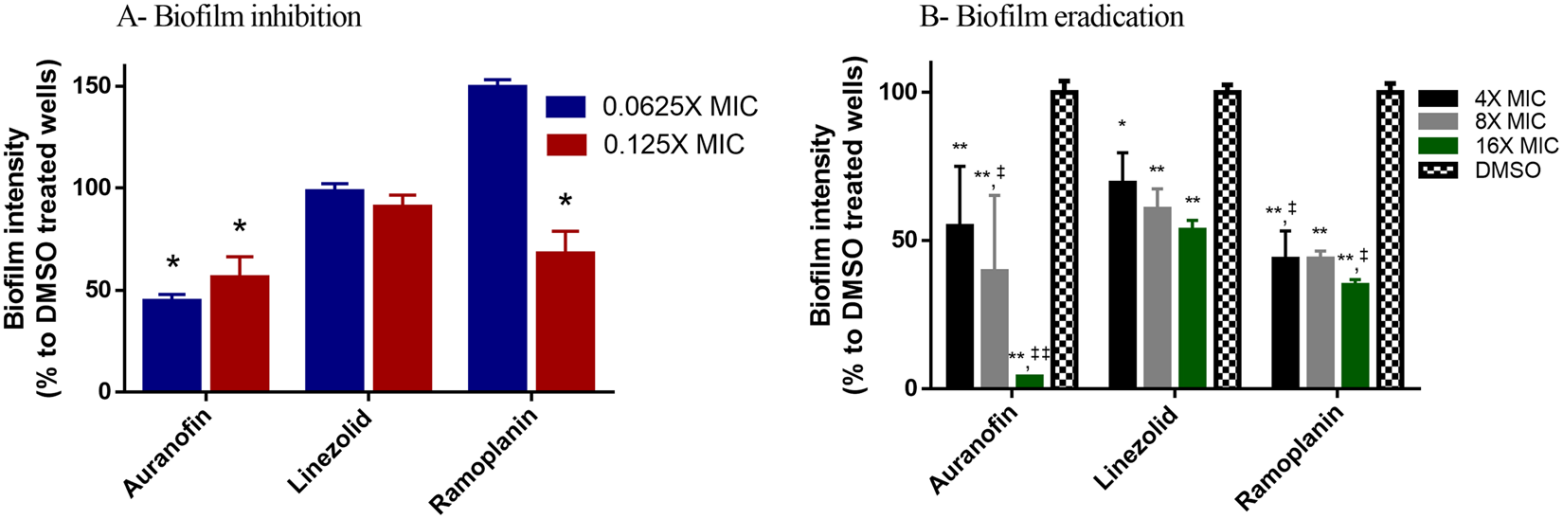
The anti-biofilm activity of auranofin against *E*. *faecalis* NR 31972. (**A**) Biofilm inhibition activity of auranofin; Sub-inhibitory concentrations of the drugs were added at the same time with the bacteria in TSB + 1% glucose and incubated for 24 hours at 37 °C, then the biofilm density was measured using crystal violet. (**B**) Biofilm eradication activity of auranofin; The bacteria were incubated for 24 hours in TSB + 1% glucose to allow for the formation of mature biofilm. Drugs were then added and incubated with the bacterial biofilm for additional 24 hours before the biofilm density was measured. (*) Denotes significant difference from the DMSO treated control, while (‡) denotes a significant difference from the linezolid treated wells at equal concentration.

To examine whether the potential therapeutic application of auranofin could be expanded beyond the inhibition of planktonic VRE, we tested the ability of auranofin to inhibit biofilm formation in VRE and remove established biofilm. As presented in Fig. 3, auranofin at subinhibitory concentrations (0.0625X and 0.125X MIC) resulted in a significant reduction (~50%) of the VRE’s biofilm-forming ability. Linezolid did not inhibit the biofilm formation in VRE, and ramoplanin exhibited a 30% VRE reduction at 0.125X MIC.

Next, we tested auranofin activity against established VRE biofilm. Auranofin at concentrations of 4, 8, and 16X MIC significantly reduced the established VRE biofilm by 50, 60 and 95%, respectively. Linezolid at 4, 8, and 16X MIC reduced the established VRE biofilm by 30, 40 and 50%, respectively. Ramoplanin concentrations of at 4, 8, and 16X MIC reduced the established VRE biofilm by 55, 55, and 65%, respectively.

### Activity of auranofin against enterococci in an *in vivo* model of intestinal VRE colonization

**Figure 4:**
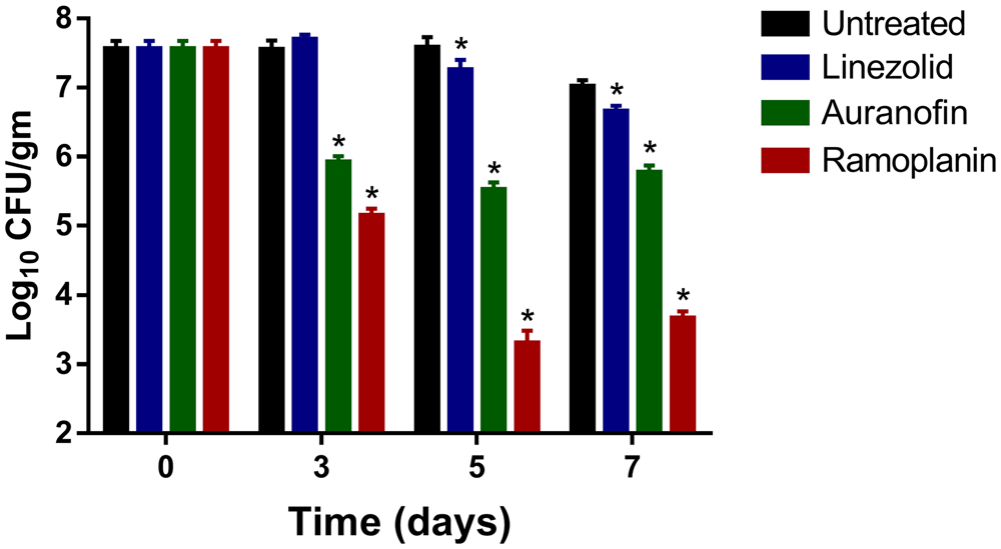
Bacterial counts of *E*. *faecium* HM-952 in the fecal samples of the mice. Infected mice were orally treated with auranofin (0.5 mg/kg), linezolid (10 mg/kg) and ramoplanin (10 mg/kg) daily for 8 days, one group was left untreated. Fecal samples were freshly collected from each group in days 0, 3, 5 and 7 post treatment. (*) Denotes significant difference from the untreated group (P < 0.05).

**Figure 5:**
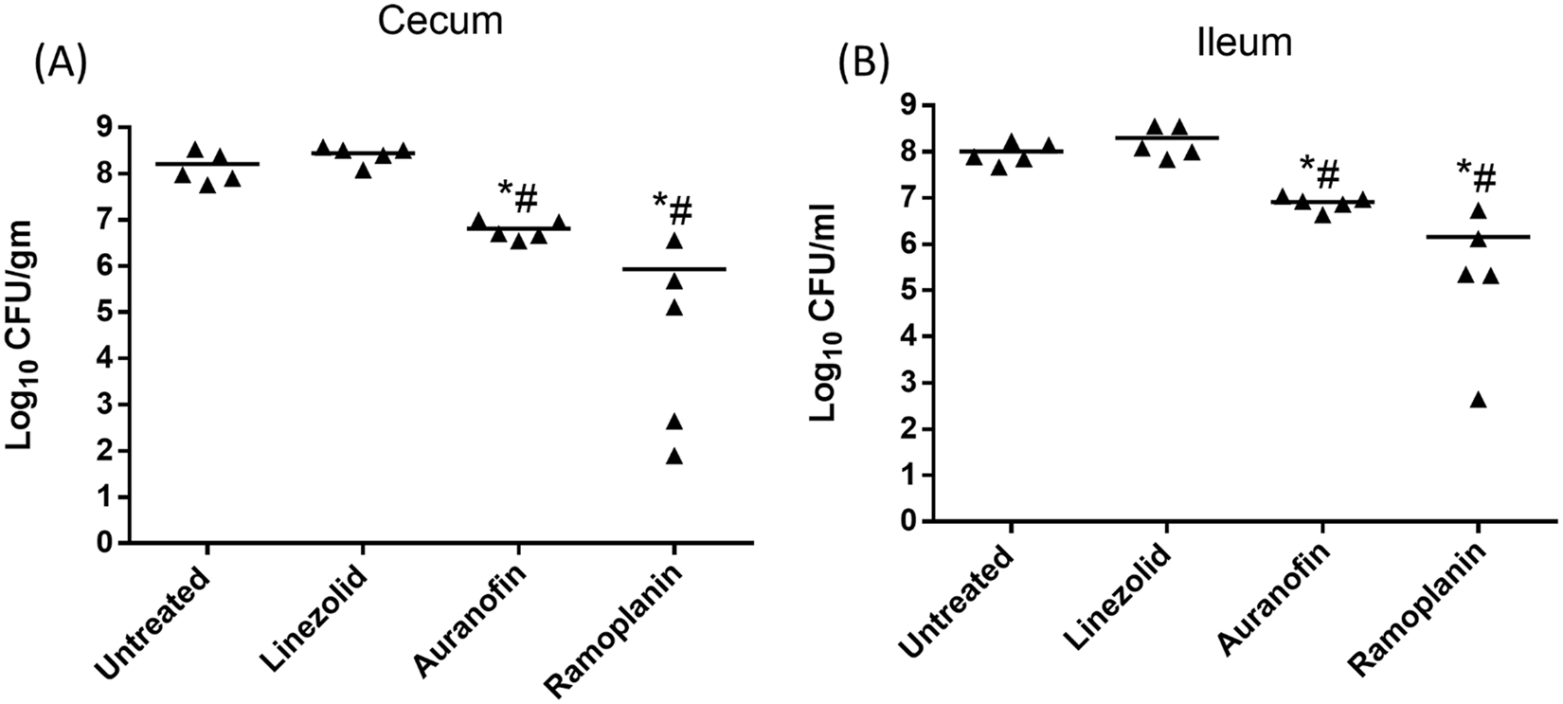
Bacterial counts of E. faecium HM 952 in (**A**) cecum and (**B**) ileum contents of the mice. Infected mice (5/group) were orally treated with auranofin (0.5 mg/kg), linezolid (10 mg/kg) and ramoplanin (10 mg/kg) daily for 8 days, one group was left untreated. Cecum and ileum contents were collected on day 9. (*) Denotes significant difference from the untreated group (P < 0.05) (#) denotes significant difference from the Linezolid-treated group (P < 0.05).

In order to validate our *in vitro* results, the VRE-colonization model^3,22,23^ was utilized to assess the ability of auranofin to reduce the shedding and burden of VRE in the gastrointestinal tract of mice. As presented in Fig. 4, auranofin and ramoplanin were superior to linezolid in decreasing the burden of VRE in fecal samples collected from mice. After three days of treatment, auranofin significantly reduced the burden of VRE in fecal samples by more than 97.79%, a rate similar to ramoplanin (99.5%). Linezolid, in contrast, was unable to reduce the burden of VRE after three days of treatment. The presence of VRE in the fecal samples of mice treated with auranofin diminished to a remarkable 99.12% reduction after five days of treatment. Linezolid generated a 52.18% reduction in the VRE CFU count of fecal samples after five days of treatment. Mice treated with ramoplanin exhibited a 99.99% reduction of VRE in the fecal samples after five days for treatment. The burden of VRE (~10^7^ CFU/gram feces) remained consistent in the control group (untreated mice) throughout the course of even days, which suggests that the decrease in VRE burden observed in mice receiving auranofin, ramoplanin, or linezolid was primarily due to the treatment received (rather than the excretion elimination of bacteria from the intestinal tract), data are shown in Fig. 5.

A similar pattern was observed in the cecum and ileum contents. Linezolid did not reduce the cecum and ileum bacterial counts. Auranofin and ramoplanin significantly reduced the VRE load in the cecum and ileum. Auranofin decreased the burden of VRE in the cecal content by 1.4-log10 and in the ileal content by 1.3-log10, relative to the untreated mice. Ramoplanin decreased the burden of VRE in the cecal content by 2.3-log10 and in the ileal content by 1.8-log10, relative to untreated mice.

## Discussion

*Enterococci* are normal inhabitants of several human body niches, especially the GIT. *Enterococci* have the capacity to overgrow other normal flora and colonize the gut, particularly after the administration of broad spectrum antibiotics. Heavy VRE gut colonization usually precedes infections and is considered the initial source of bacteremia-induced endocarditis^2,3,24,25^. Moreover, the increased number of colonized patients increases the colonization pressure and the enterococcal acquisition by other patients^26^. *Enterococci* are naturally resistant to several antibiotics and can rapidly develop resistance to antibiotics via several mechanisms^27,28^. Vancomycin resistance is one of the most remarkable resistances developed by *enterococci*. More than one third of enterococcal infections in the U.S. are caused by VRE, and the percentage is higher for infections caused by *E*. *faecium* (almost 80%)^29^. Furthermore, VRE also displays resistance to the other clinically important antibiotics like ampicillin and aminoglycosides^2,28,30^. *Enterococci* are capable of forming biofilm, a feature that not only facilitates infections but also helps to colonize the GIT^31,32^.

Despite the significance of enterococcal colonization of the gut, the FDA has yet to approve a drug that can be used for VRE decolonization^2,25^. Also, the current control methods, including contact precautions and isolation of the patient are costly, have negative psychological impact on the patients and were not proven to have positive clinical outcomes^33^. Overall, there is an unmet and urgent need for a potent decolonizing agent against VRE.

Auranofin is an FDA-approved drug for the treatment of rheumatoid arthritis. It has a well-defined toxicity profile and an acceptable safety for human use^34,35^. Auranofin was reported to have a potent inhibitory activity against Gram-positive bacteria, including *enterococci*, fungi and parasites^12,36,37^. Due to its poor intestinal absorption auranofin has been granted orphan-drug status from the FDA for treatment of intestinal amebiasis, and is currently in a Phase II clinical trial for treatment of intestinal giardiasis^16,34,36^.

In the current study we showed the superior *in vitro* activity of auranofin against a broad panel of *enterococcus* isolates compared to linezolid (the drug of choice for the treatment of VRE infections) and ramoplanin (clinical molecule currently in clinical trials for VRE decolonization). It is worth mentioning that the activity of auranofin did not change between vancomycin-resistant and vancomycin-sensitive strains. Auranofin was also consistent against both *E*. *faecium* and *E*. *faecalis*, a property that is lacking in some current antibacterial agents, for example quinupristin-dalfopristin^27^.

A time-kill assay was utilized to ascertain whether auranofin is bacteriostatic or bactericidal. Auranofin exerted a bacteriostatic effect against the tested VRE isolate, a result that concurs with the previous report against *staphylococcus aureus* at equal concentration^38^. Although the bacterial count was reduced after 24 hours, it did not reach to the 3-log10 cutoff value that distinguishes bacteriostatics from bactericidals. The bacteriostatic activity of auranofin should not be discouraging, especially in the GIT environment of numerous competing microorganisms.

In previous studies, auranofin exhibited a complex mechanism of action against methicillin-resistant *staphylococcus aureus* (MRSA), a possible reason for MRSA’s inability to develop resistance against auranofin^36^. These results were encouraging for our test of VRE’s ability to develop resistance against auranofin. As reported earlier, the MIC of auranofin against the tested isolate did not change during the 14-day experiment and even up to 25 days (data not shown). This is in agreement with the previous reports of failure to generate auranofin-resistant MRSA^36,39^.

As discussed earlier, biofilm is a virulence factor that helps enterococci to establish colonization. Biofilm-positive enterococcal phenotypes are associated with GI colonization. Auranofin is known to inhibit the enterococcal biofilm through the inhibition of selenium metabolism and selenoenzymes, given that biofilm formation is reported to enhance gut colonization of *enterococci*^31,40^. We sought to test the biofilm inhibition activity of auranofin as well as the ability of auranofin to eradicate established biofilms. As was expected, when auranofin was incubated with the bacteria, auranofin significantly inhibited the biofilm formation at sub-MIC concentrations (Fig. 3A). While linezolid and ramoplanin had no effect at all or a minimal effect, auranofin inhibited about 50% of the biofilm formation when compared to the untreated control. Additionally, auranofin treatment to the already formed biofilm drastically reduced the biofilm intensity at supra-MIC concentration. This effect was superior to those of linezolid and ramoplanin (Fig. 3B). Biofilm enhances enterococcal colonization capacity, and the antibiofilm activity is particularly important in evaluating the decolonization efficiency of auranofin^31^.

Auranofin’s potent *in vitro* activity against numerous multi drug-resistant enterococcus strains and the inhibition of VRE biofilm (combined with the low intestinal absorption of auranofin and the inability of VRE to develop resistance against auranofin *in vitro*) prompted us to investigate the efficacy of auranofin *in vivo* in the VRE colonization mouse model. We were interested in evaluating the ability of auranofin to reduce the VRE shedding in fecal samples as well as reduce the VRE burden in the mice guts. The ampicillin-primed mice were infected with a VRE strain, and treatment began after the VRE colonization was established. At a concentration of 0.5 mg/kg, auranofin significantly reduced the bacterial shedding in fecal samples after only three days of treatment. By the seventh day of treatment, the bacterial shedding was reduced by about 99% compared to the untreated control (Fig. 4). Auranofin (at the same concentration, 0.5 mg/kg) reduced the bacterial load in cecum and ileum contents (Fig. 5) by more than 99.9%. This effect was superior to linezolid at a concentration of 10 mg/kg, which had a minimal effect in fecal samples and no significant effect in cecum and ileum contents. Although both auranofin and linezolid are bacteriostatic, linezolid’s lack of activity is possibly due to its complete absorption from the GI tract^41^. The auranofin dose used in this study is lower than the published toxic dose (TD_Lo_) in humans (0.54 mg/kg)^42^, and is far less than the oral LD50 in mice, 84.94 mg/kg^43^. Although auranofin was not as effective as ramoplanin in reducing the VRE burden in the mouse model, auranofin is FDA-approved and ramoplanin—a Phase II clinical molecule—is not. Further investigations are needed to test whether VRE recurrence observed with ramoplanin treatment is also encountered with auranofin treatment^7^ as well as the effect of auranofin on the normal bacterial population of the gut. Overall, the current study suggests that auranofin is a good candidate for further investigation as a decolonizing agent of VRE.

## Materials and Methods

### Bacterial strains and chemicals

Vancomycin-resistant *Enterococci* (VRE) strains (Table 1) were obtained from the American type culture collection (ATCC) and Biodefense and Emerging Infections Research Resources Repository (BEI Resources). All experiments were carried out in accordance with relevant guidelines and regulations and were approved by the Institutional Biosafety Committee of Purdue University. All chemicals and reagents were purchased from commercial vendors. Auranofin, linezolid (Chem-impex International, Wood Dale, IL), ampicillin (Peosta, IA), vancomycin hydrochloride (Gold Biotechnology, St. Louis, MO), gentamicin sulfate (Fisher Bioreagents, Fairlawn, NJ), and ramoplanin (Sigma-Aldrich, St. Louis, MO) were purchased commercially. Brain heart infusion (BHI), tryptic soya broth (TSB), tryptic soya agar (TSA) and enterococcosel broth were purchased from BD (Becton, Dickinson and Company, Cockeysville, MD) and Phosphate buffered saline (PBS) was purchased from corning (Corning, NY).

### *In vitro* antibacterial assay

The standard broth microdilution assay was utilized to assess the minimum inhibitory concentration (MIC) of auranofin and the control antibiotics following the guidelines of the Clinical and Laboratory Standards Institute (CLSI)^44^. The minimum inhibitory concentrations (MICs) reported are the lowest concentration of each drug that could inhibit the visual turbidity due to bacterial growth. MIC_90_ is the drug concentration that can inhibit the growth of 90% of the tested isolates.

### Killing kinetics of auranofin and control drugs

A time-kill assay was performed as described previously^45–48^. Briefly, an overnight culture of *E*. *faecium* HM-952 was diluted to approximately 10^6^ CFU/ml. Three- and six-fold MIC of auranofin and the control drugs, in triplicates, were incubated with the bacterial suspension for 24 hours. At the indicated time intervals, samples were taken, diluted, and cultured on BHI agar to detect the bacterial counts at each time point.

### *In vitro* development of resistant mutants

To assess the propensity of VRE to develop resistance against auranofin, *E*. *faecium* strain HM-952 was serially passaged with sub inhibitory concentrations of auranofin and control drugs (linezolid, ramoplanin, and gentamicin) in 14 passages over a period of two weeks. Resistance was defined as a 4-fold shift in the MIC^20,21,23^.

### Auranofin activity against VRE biofilm

A Biofilm Inhibition assay was utilized to examine the effect of sub-inhibitory concentrations of auranofin on the ability of VRE to form biofilm as described before^49,50^. Briefly, an overnight culture of *E*. *faecalis* NR 31972 was diluted 1:100 in TSB +1% glucose and seeded in 96-well plates. Different sub-inhibitory concentrations of auranofin and control antibiotics (linezolid and ramoplanin) were added, and the plates were incubated for 24 hours at 37 °C. After incubation, the medium containing drugs and planktonic bacteria was discarded, and the adherent biofilms were washed twice with Phosphate buffered saline (PBS). The plates were then stained with 0.1% crystal violet for 30 minutes and washed again to remove the non-adherent stain. The remaining stain was solubilized using 95% ethanol for 45 minutes. Then the OD595 was measured using a kinetic microplate reader (SpectraMax i3x, Molecular Devices LLC, Sunnyvale, CA).

The auranofin activity was assessed against the mature biofilms using the aforementioned protocol. The drugs were added at concentrations higher than the MIC to allow for the effective eradication of the mature biofilm. All experiments were carried out in quadruplicates and repeated at least twice.

### Decolonization of VRE from the gastro-intestinal tract of mice

The study was reviewed, approved, and performed under the guidelines of the Purdue University Animal Care and Use Committee (PACUC) and carried out in strict accordance with the recommendations in the Guide for the Care and Use of Laboratory Animals of the National Institutes of Health. To assess the efficiency of auranofin in decolonizing VRE from the gut in an animal model, the VRE mice decolonization model was followed^3,23^. Briefly, 6-week-old female C57BL/6 mice (Envigo, Indianapolis, IN) were sensitized by ampicillin (0.5 gm/ml in drinking water) for seven days. One day later mice were orally infected with 3 × 10^8^ CFU/mL of VRE strain *E*. *faecium* HM-952. Seven days post infection, the mice were divided into groups and treated orally with either auranofin (0.5 mg/kg), linezolid (10 mg/kg), ramoplanin (10 mg/kg), or PBS. Treatments were continued for eight days before the mice were humanely euthanized on day 9 via CO_2_ asphyxiation. Fecal samples were taken freshly from the mice on days 0, 3, 5 and 7 post treatment. The cecum and ileum contents were collected following euthanasia. The contents of the cecum and ileum as well as the fecal sample were weighed, diluted with PBS, plated on enterococcosel agar (supplemented with vancomycin, 8 µg/mL), and incubated for 48 hours at 37 °C to determine the bacterial count in each sample.

### Statistical analysis

GraphPad Prism version 7.00 for Windows (GraphPad Software, La Jolla CA) was utilized in performing the statistical analysis. Two-way ANOVA followed by Dunnett’s multiple comparisons test was performed to analyze biofilm inhibition and eradication data and the data from fecal samples. For the data generated from cecum and ileum content, one-way ANOVA was used and was followed by t test for the post hoc pairwise comparison.

### Data availabilit

All data generated or analyzed in this study are available from the corresponding author on reasonable request.
